# Removal of Ingested Magnetic Bodies via Laparoscopic Appendectomy

**DOI:** 10.1055/s-0040-1714669

**Published:** 2020-10-21

**Authors:** Vojtech Dotlacil, Barbora Frybova, Martin Vyhnanek, Lubos Zeman, Michal Rygl

**Affiliations:** 1 Department of Paediatric Surgery, Second Faculty of Medicine, Charles University and Motol University Hospital, Prague, Czech Republic

**Keywords:** magnet ingestion, laparoscopic appendectomy, pediatric, surgery, hypochondrium

## Abstract

Ingestion of a foreign body is a frequent diagnosis in the pediatric population. In a small percentage of cases, foreign bodies themselves are strong magnets, and swallowing of multiple magnetic bodies can lead to serious complications in the gastrointestinal tract. Two consecutive case reports of patients who swallowed two magnetic beads are presented. In both cases, the abdominal radiograph described two magnets in contact, one in the area of the left hypochondrium and one in the right hypogastrium. Attempts of endoscopic localization and removal were unsuccessful. Due to the failure of magnet progression, laparoscopic revision of the abdominal cavity was indicated in both patients on the 25th and 4th day after swallowing. Using the magnetic forces between the magnets and the laparoscopic instruments, the foreign bodies were localized in the appendix of the first patient and in the cecum of the other one. The magnets were extracted together with the removal of the appendix in both patients. This is one of the first articles describing the successful extraction of foreign magnetic bodies from the gastrointestinal tract via laparoscopic appendectomy.

## New Insights and the Importance for the Pediatric Surgeon

An elegant and simple minimally invasive surgical method how to extract nonprogressive multiple magnetic foreign bodies form gastrointestinal tract without the need of enterotomy.

## Introduction

Swallowing of a foreign body (SFB) is a frequent diagnosis in the pediatric population, with the highest incidence in the age category from 6 months to 6 years. Only 1% of patients require surgical intervention. Among the SFBs, specific properties lead to the special position of small SFB composed of powerful magnets (high-powered neodymium) that are used in different types of toys. Their ingestion and possible interaction across the intestinal wall pose a risk of development of severe gastrointestinal complications such as pressure necrosis of the intestinal wall, perforation, fistulas, bleeding, or intestinal obstruction. 1 2 This report describes a technique for the extraction of foreign magnetic bodies from the gastrointestinal tract by means of laparoscopic appendectomy.

## Case Reports

### Case no. 1

**Fig. 1:**
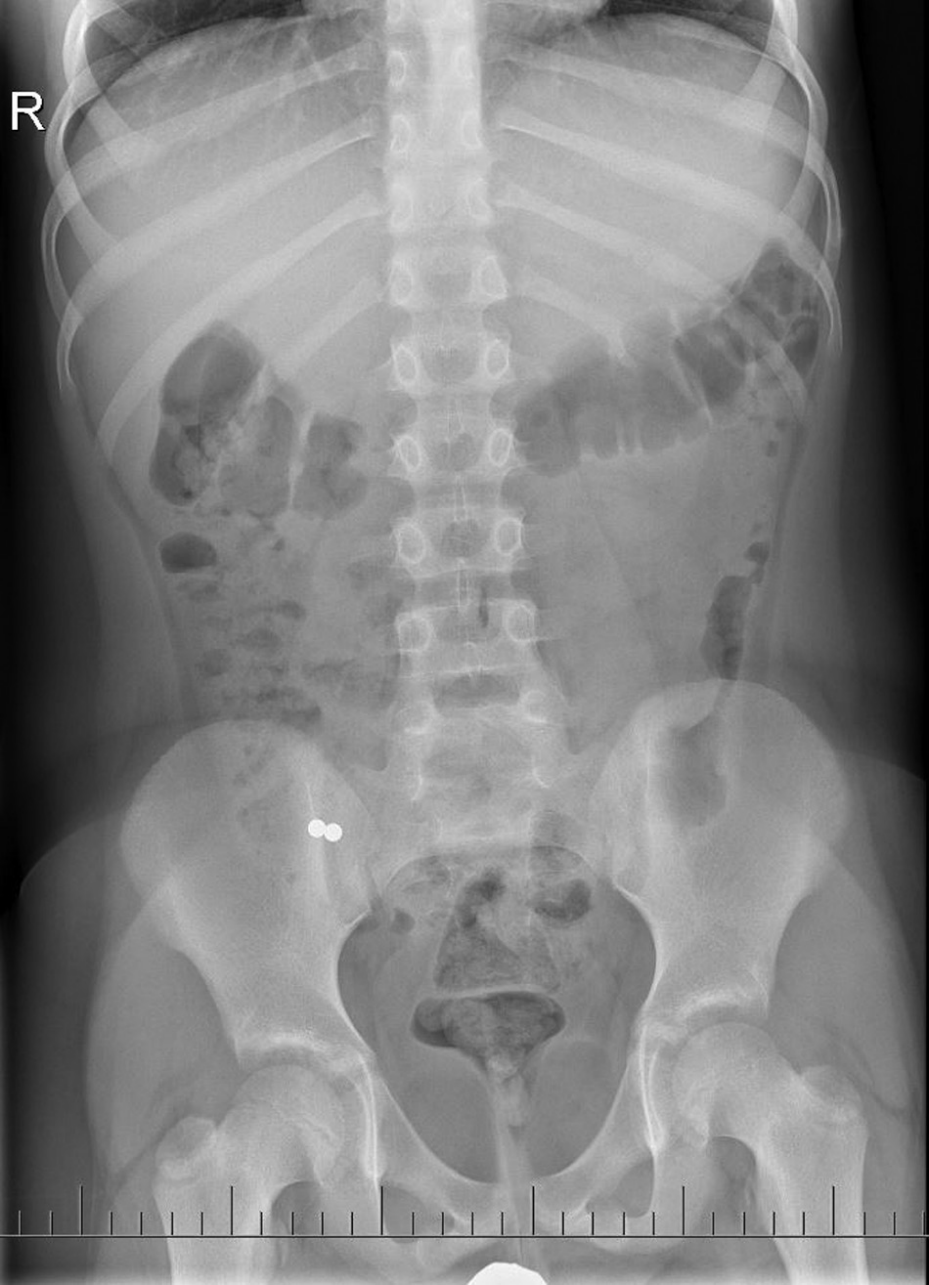
The location of MFBs on an abdominal X-ray native abdominal image. MFBs, magnetic foreign bodies.

A 9-year-old boy was admitted with a history of swallowing magnetic foreign bodies (MFBs) 3.5 hours before admission. Upon admission, the boy was free of complaints; his physical and laboratory testing were without any significant details. An abdominal X-ray examination showed two oval MFBs adjacent to one another, 5 mm in size, in the left hypochondrium. Because of the possible localization of the MFBs in the stomach, upper gastrointestinal endoscopy was performed after admission, but the MFBs were not found in the stomach. Following control X-ray examinations on days 1, 3, and 10, the foreign bodies were found to have remained in a stationary position in the right hypogastrium on day 10 ( Fig. 1 ). On the day 10, colonoscopy to the terminal ileum was performed after bowel preparation (Macrogolum 4000) but the MFBs were not found. Due to the patient's well-being, a conservative outpatient regime was chosen. Due to the fixed position of MFBs, on the day 25 after swallowing, the patient was indicated for laparoscopic abdominal cavity revision.

### Case no. 2

**Fig. 2:**
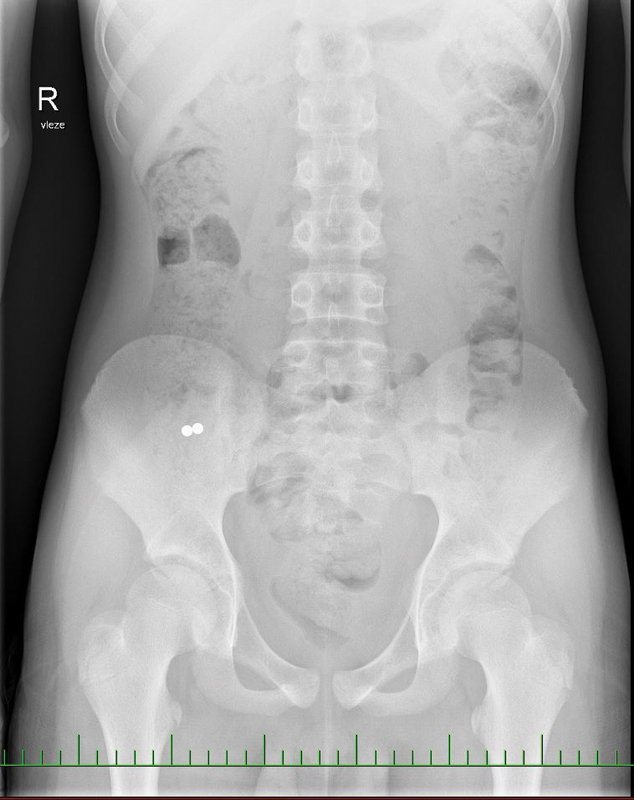
Abdominal X-rays contrasting oval MFBs in the right hypogastrium. MFBs, magnetic foreign bodies.

**Fig. 3:**
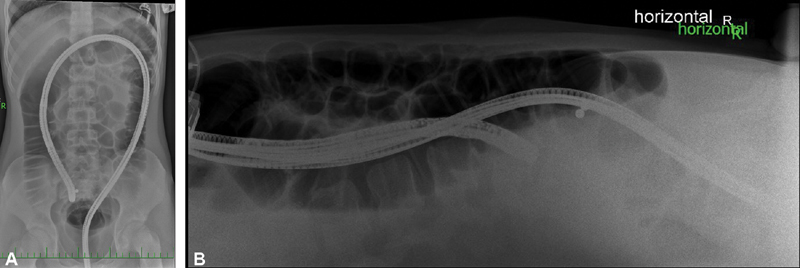
( **A, B** ) Colonoscopy under skiascopic control with visible MFBs. MFBs, magnetic foreign bodies.

A 13-year-old boy with a 6-hour history of swallowing two MFBs was admitted for hospitalization. Both the clinical and laboratory examinations were within expected limits. Abdominal X-ray imaging showed two spherical foreign bodies in tight contact projected into the right hypogastrium ( Fig. 2 ). Colonoscopy and esophagogastroduodenoscopy examinations were performed the next day, after bowel preparation (Macrogolum 4000). The MFBs were not found by any of these methods despite the fact that endoscopies were performed under skiascopic control. The MFBs were displayed in the right hypogastrium, but out of range of the endoscope ( Fig. 3A, B ). The patient had no clinical difficulties. Due to the fixed MFB position on repeated X-ray examinations, laparoscopic revision of the abdominal cavity was indicated on the day 4 after swallowing the MFBs.

### Operative Technique

**Fig. 4:**
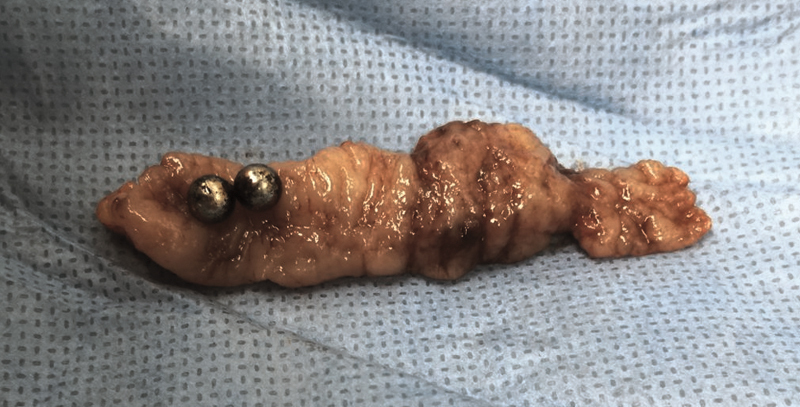
Perioperative finding: an incised appendix vermiformis containing two MFBs. MFBs, magnetic foreign bodies.

A standard three-port laparoscopic appendectomy was performed in both cases (10-mm port in the umbilicus for the camera, 5-mm port in the left mesogastrium, and 5-mm port 2 cm above the symphysis in the midline). During the laparoscopic revisions, the MFBs were found in the appendix in case no. 1 and in the small bowel in case no. 2 using the magnetic forces between the metal end of the grasper and the MFBs. The MFBs in case no. 2 were pushed into the appendix using the magnetic attraction of the metal parts. Subsequently, standard laparoscopic appendectomies were performed with the removal of the MFBs in the lumen of the appendix which was closed by Vicryl endoloop (Johnson & Johnson Medical N.V., Belgium) via the extractor in the umbilical port ( Fig. 4 ). Macroscopically, the appendixes appeared without inflammation and histological examination did not show pathological changes. Both postoperative courses were uneventful and the patients were discharged on the postoperative day 3.

## Discussion

MFBs account for only 3% of the total number of swallowed foreign bodies. 3 However, SFBs made of strong magnets represent a high risk of gastrointestinal tract damage and requires an active, specific approach. In 2017, Cho et al presented a set of three patients who, after swallowing MFBs, suffered from the perforation of different gastrointestinal tract sections. However, none of the patients experienced a sudden abdominal emergency requiring an acute operation. 4

The specific properties of MFBs, the increasing incidence of their ingestion and possible serious complications have contributed to the creation a separate care algorithm. This algorithm was published by Sola et al in 2018 and suggested that the most endangered group of patients are those who swallow multiple MFBs or have a combination with of other magnetizable objects that should be observed during a hospitalization with repeated clinical and X-ray examinations followed by an endoscopic and/or surgical intervention. 5

Surgery is the method of choice if MFBs lead to clinical problems or if they cannot be removed endoscopically, as well as in cases where there is a failure of magnet progression after 48 hours. In most of these patients, the laparoscopic approach is feasible and safe, which was confirmed in our case reports. Moreover, during the laparoscopy, the magnetic forces between the laparoscopic instruments and the MFBs can be used for better localization. In the literature, only a few studies have described extraction through laparoscopy. 6 7 8

Following an unsuccessful attempt at endoscopic removal, laparoscopy is a suitable method for nonprogressive multiple MFBs in patients without clinical symptomatology. If it is possible, removal of the MFBs together with the appendix is technically simple and incurs less risk compared with extraction via enterotomy.
